# Critical field of 2H-NbSe_2_ down to 50mK

**DOI:** 10.1186/2193-1801-3-16

**Published:** 2014-01-09

**Authors:** Adel Nader, Pierre Monceau

**Affiliations:** Atomic Energy Commission of Syria, Department of Physics, P.O Box 6091, Damascus, Syria; Institut Néel CNRS/UJF UPR2940, 25 rue des Martyrs, BP 166, Grenoble, Cedex, 38042 France

**Keywords:** *C*ritical fields, 2H-NbSe_2_, *L*ayered superconductor

## Abstract

Critical field of 2*H*-NbSe_2_ is determined for the field perpendicular to the conducting planes down to 50 mK, by magnetoresistance measurements. It is the first time that such a measurement is extended below 1 K. Variations are almost linear down to 1 K, with a little upward curvature, and the slope of *H*_*c* 2⊥_ (*T*) decreases below 1 K. The reduced critical field extrapolates to 0.9 when the temperature approaches zero, higher than the WHH upper limit of 0.69 conformably with the extension of this model for anisotropic superconductors.

Angular dependence of the critical field is also determined at 5.5 K. Variations are the same as expected for a 3D-anisotrpic superconductor and the anisotropy value confirms previous results.

## Introduction

The structure of 2*H*-NbSe_2_ consists of three-layer packets, inside which the layers are ordered in the Se-Nb-Se sequence with covalent binding between them, whereas the packets are bound by van der Waals coupling. Due to this weak coupling in the dichacogenides family, adjacent packets may be oriented in different ways relative one to another and as a consequence the possibility of polytypism. The most widely encountered poly-type for NbSe_2_ is the 2*H*, where 2 is the number of packets in the unit cell and *H* stands for hexagonal. The structure of 2*H*-NbSe_2_ was reviewed by Meerschaut and Deudon (2001).

This compound has been extensively studied for its superconductivity and the formation of a CDW state. It shows a superconducting transition at 7.2 K (Toyota et al. 1976 Sanchez et al. 1995 Soto et al. 2007) with an upper critical magnetic field anisotropy, induced by the layered structure, of about 3.

The CDW state appears in 2*H*-NbSe_2_ below *T*_CDW_ ~ 33 K (Moncton et al. 1977 Higemoto et al. 2003). It can be seen as an anomaly in the resistivity curve that the amplitude is tightly proportional with the sample purity (Iwaya et al. 2003)

An interesting aspect in the superconducting state of this compound which still attracts lots of attention is the peak effect (Banerjee et al. 1998). This effect appears as a maximum in the critical current curve in function of temperature.

Another interesting aspect in this compound is the two gaps structure (Huang et al. 2007 Rodrigo and Vieira 2004) which was evidenced, mainly, by specific heat measurement under magnetic field.

Though, it has been believed for a long time that 2H-NbSe_2_ has just an anisotropic superconducting gap and some experimental results were explained into this framework (Toyota et al. 1976)

The behavior of the critical magnetic field has been subject to discussion since the seventies since its behavior depends tightly on the Fermi Surface geometry. As a result many theoretical calculations (Werthamer et al. 1966 Arai and Kita 2004) concluded that the critical field perpendicular to the layers vary linearly near T_c_ and its slope decreases at lower temperatures. Though an upward curvature is expected for the critical field in the parallel direction (Arai and Kita 2004).

The perpendicular critical field saturation at very low temperatures has never been investigagted experimentally.

In this work critical magnetic field of this compound is determined by mean of magneto-resistance measurement down to 50mK for the first time. It shows a behavior as expected by theoretical models mentioned above with almost a linear variations near T_c_ and a slope decreas at very low temperature. Angular dependence of the critical field is also determined at 5.5 K, it confirms previous results.

## Experimental details

The NbSe_2_ powder is prepared starting from constituting elements at stoechiometric ratio, and then the crystals are grown by thermal gradient using the iodine as a transport agent. Single crystals of these compounds are in platelet form with a diameter in the range of 2 mm and a thickness of 50 μm.

Measurements down to 50 mK are done using a top loading dilution refrigerator and a 8 Tesla superconducting magnet. The anisotropy is measured at 5.5 K using a rotating system with an accuracy of 0.1°.

## Results and discussion

**Figure 1:**
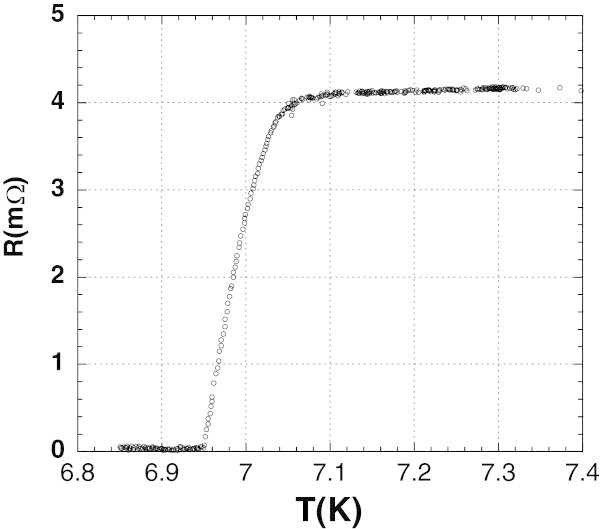
**The superconducting transition of 2H-NbSe**_**2**_ **.** T_c_ was taken at half height of normal state resistance.

Our best sample, of the sharpest transition has a critical temperature at half height of normal state resistance of 7.0 K, with a transition width of about 0.8 K, as shown in Figure 1.

**Figure 2:**
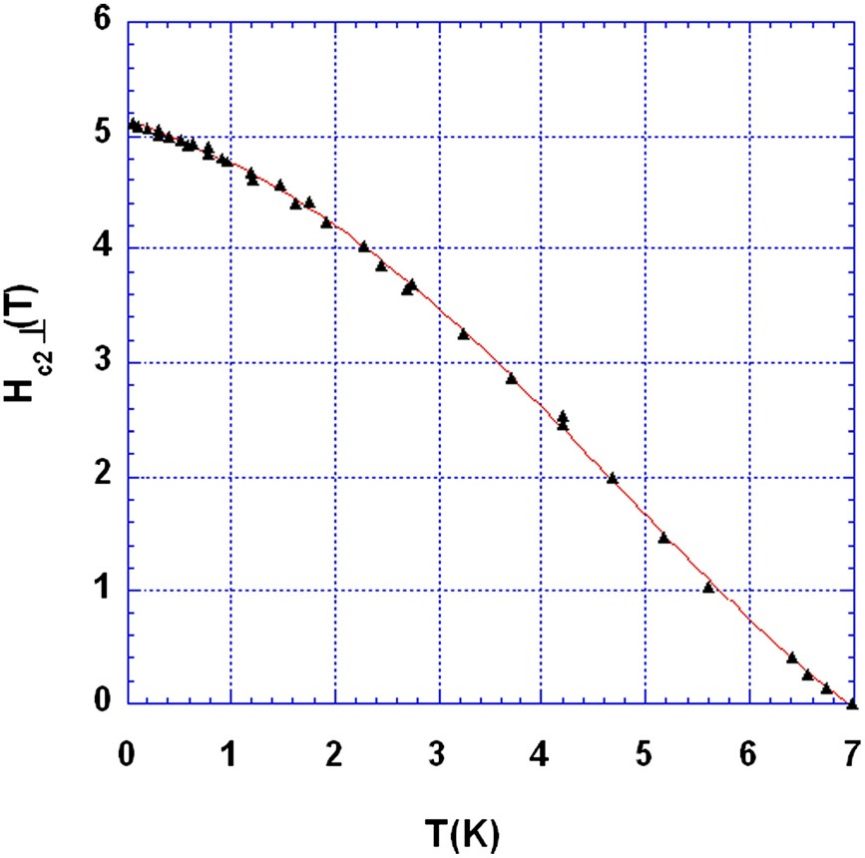
**Critical magnetic field down to 50 mK.** Continuous line is a guide for eye. Note the saturation below 1 K.

The upper critical magnetic field is taken at half height of the measured magneto-resistance. Figure 2 shows variations of *H*_*c* 2⊥_ (*T*). It shows a little upward curvature near Tc. Though, by experience, upward curvature near T_c_ may be induced by a rather wide resistive transition, a better discussion of such an aspect would then need a better quality sample, with a sharpest transition. Nevertheless, such an upward curvature was expected by by Arai and Kita (2004) for the parallel critical field or for layer compounds of higher anisotropies (Woollam et al. 1974).

Below 1 K the slope of *H*_*c*__2⊥_ (*T*) decreases as expected by Werthamer et al. (1966) for a dirty superconductor and by Arai and Kita (2004)).

Our results are similar to that found in (Toyota et al. 1976) where the measurements were done only down to 1.3 K. Though, no upward curvature near T_c_ was mentioned in (Toyota et al. 1976), probably because samples were of a much better quality; their RRR ratio was always higher than 30, although, for our sample it is less than 10.

**Figure 3:**
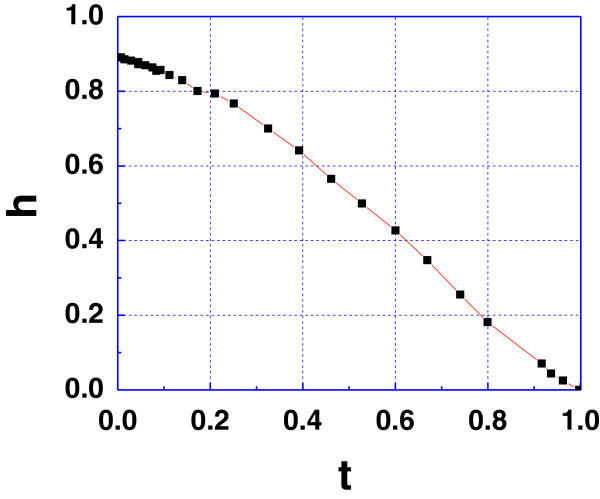
**Critical magnetic field in reduced coordinates.** Continuous line is a guide for eye Note the value of *h* (0).

Figure 3 shows *h* (*t*), which is the upper critical field normalized as in (Werthamer et al. 1966) i.e. *t* = *T* /*T*_*c*_ and *h* = *H*_*c*2_ /(-*dH*_*c* 2_/*dt*). *h* (*t*) extrapolates to 0.9 when *t* → 0.

Werthamer et al. (1966) extended the calculation of the upper critical field for a bulk superconductor by taking in account Pauli spin paramagnetism and spin-orbit impurity scattering. According to this model this value should not be greater than 0.69. Though, in (Hohenberg and Werthamer 1967) it was shown that this is possible for anisotropic superconductors when Fermi surface is no more spherical.

Also, the sketch of *h (t)* seems to be in good agreement with the reduced critical field curve calculated using the Fermi surface obtained by local density approximation (LDA) by Arai and Kita (2004) (see Figure 1 in this reference). According to this calculation *h* (0)extrapolates to about 0.95.

**Figure 4:**
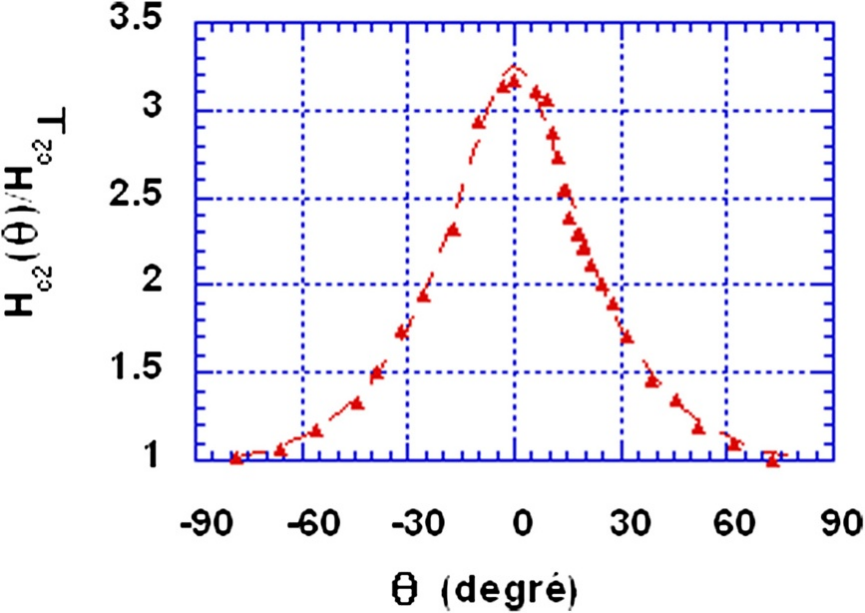
**Critical magnetic field in function of the angle between conducting planes and magnetic field at 5.5 K.** Continuous line is the fit with Lawrence and Doniach expression. The anisotropy is 3.2

Figure 4 shows variations of *H*_*c*2_ (*θ*) at 5.5 K, where *θ* is the angle between the conducting planes and the magnetic field. The following formula was given by Lawrence and Doniach (1971):
1

where *ϵ* is the ratio of the parallel critical field to the perpendicular one at *T*.

The fit with this formula gives an anisotropy of 3.2. This value approaches those found in (Toyota et al. 1976 Sanchez et al. 1995).

Though, Lawrence and Doniach simple model based on the effective mass anisotropy was proven not to be sufficient to describe the critical field anisotropy in layered superconductors as shown by Toyota et al. (1976) and that other assumptions should be made such as the gap anisotropy, but it could be used as a first approximation to extract anisotropy from the *H*_*c*2_ (*θ*, *T*) curve.

Let us note also that the critical field anisotropy is temperature dependent, and the parallel critical field shows an upward curvature in (Toyota et al. 1976 Muto et al. 1973).

In plane and out-of-plane coherence lengths are then *ξ*_||_ (0) =74 Å and *ξ*_⊥_ (0) = 23 Å respectively. *ξ*_⊥_ (0) is much greater than the distance separating the centers of two successive conducting planes, which confirms that 2*H*-NbSe_2_ is a 3D-anisotropic superconductor.

Coherence lengths as well as the critical field anisotropy are of the same range as found in (Sanchez et al. 1995) by specific heat measurements.

## Conclusion

Critical magnetic field measurement of 2*H*-NbSe_2_ is extended down to 50 mK. The slope of *H*_*c*__2⊥_ (*T*) decreases below 1 K and varies almost linearly near T_c_ with a little upward curvature, probably induced by a rather large superconducting transition.

Also, the anisotropy is the same as obtained in previous works.
